# Supplementary material for: Skp2 and Slug Are Coexpressed in Aggressive Prostate Cancer and Inhibited by Neddylation Blockade
Source: Int J Mol Sci. 2021 Mar 11;22(6):2844. doi: 10.3390/ijms22062844 (PMC8000894; doi:10.3390/ijms22062844)
Supplement: Supplementary file 1 [file ijms-22-02844-s001.pdf]

Supplementary materials

Supplementary Table S1 Primary antibodies used for immunohistochemistry.

Antibody	Clone or Cat #	Method	Dilution	Supplier
Skp2	2C8D9	Ventana Ultra	1:75	Invitrogen
Slug	C19G7	MW, citrate, pH 6.0	1:50	Cell signaling
androgen receptor	AR441	MW, citrate, pH 6.0	1:100	Dako
E-cadherin	NCH-38	MW, citrate, pH 6.0	1:50	Dako
Ki67	MIB-1	MW, citrate, pH 6.0	1:200	Dako
beta-catenin	E-5	MW, citrate, pH 6.0	1:20	Santa Cruz

MW, microwave.

Supplementary Table S2 Primary antibodies used for western blot analysis.

Antibody	Clone	Cat. No	Dilution	Supplier
Skp2	2C8D9	32-3300	1:600	Invitrogen
Slug	C19G7	9585	1:1 000	Cell signaling
Twist	2C1A	sc-81417	1:250	Santa Cruz
GAPDH	G8795	G8795	1: 10 000	Sigma
$p27^{Kip1}$	SX53G8.5	sc-53871	1:200	Santa Cruz
MdmX	8C6	04-1555	1:200	Merck Millipore
N-Cadherin	32/N-cadherin	610920	1:2500	BD Pharmingen
E-cadherin	36/E-cadherin	610182	1:5000	BD Pharmingen
FBXO11	polyclonal	A301-177A	1:2 000	Bethyl lab.
α-tubulin	DM1A	T5168	1:1000	Sigma-Aldrich
lamin A/C	EPR 4068	ab108922	1:1000	AbCam
Trop2	polyclonal	AF650	1:250	R&D
Zeb1	polyclonal	A301-922A	1:500	Bethyl lab.
vimentin	V9	V6389	1:500	Sigma-Aldrich
β-actin	AC-15	A5441	1:8000	Sigma-Aldrich
androgen receptor	N-20	sc-816	1:200	Santa Cruz

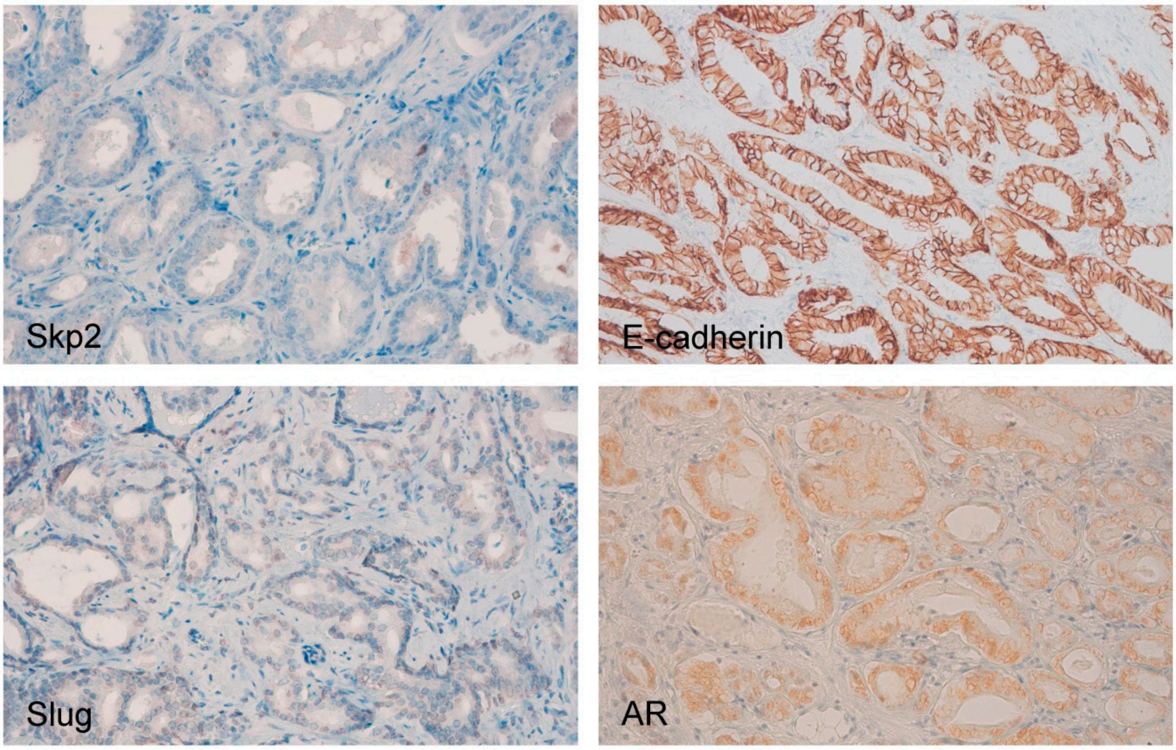

Supplementary Figure S**1.** Example of immunohistochemical staining of Skp2, E-cadherin, Slug and androgen receptor in localized primary prostate cancer tissue. All images are at magnification ×200.

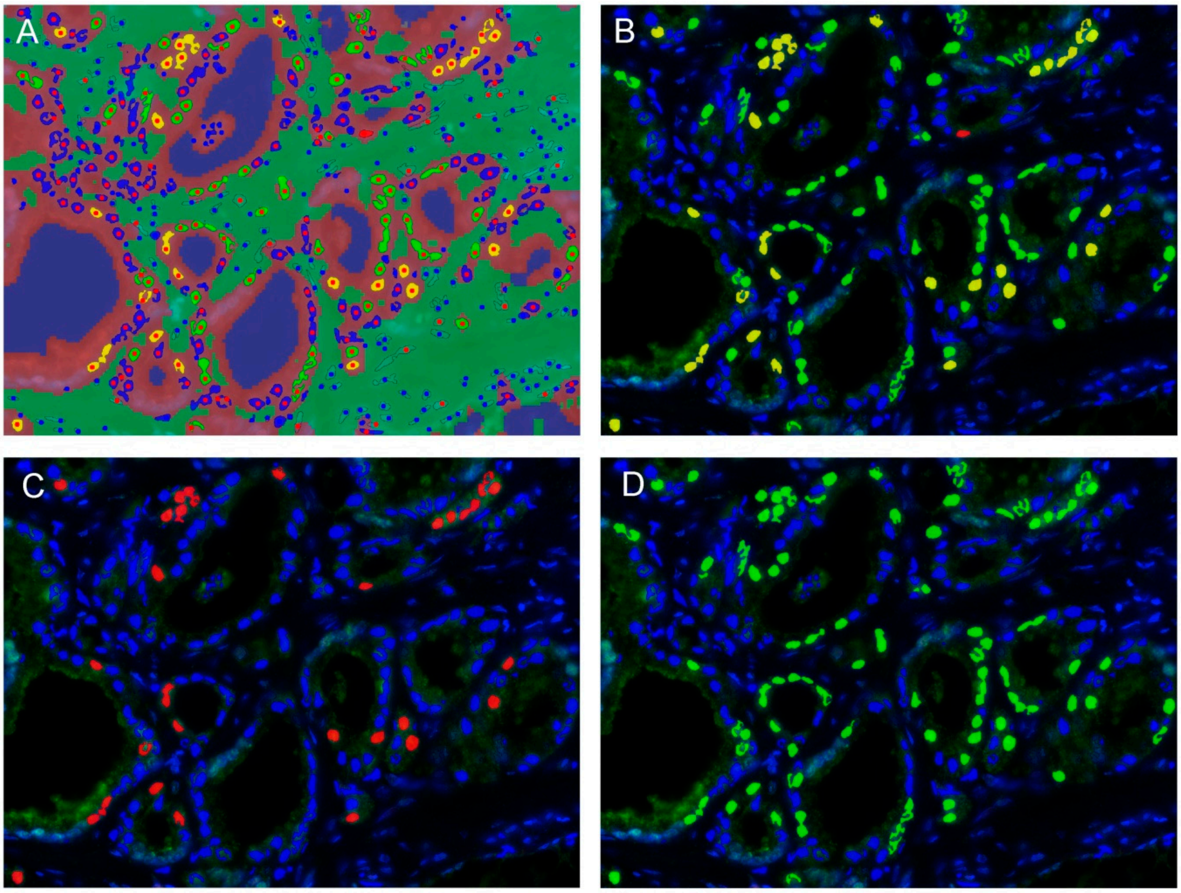

Supplementary Figure S2. Analysis of multiplex immunohistochemistry by Mantra system. (**A**) Schematic visualisation of tissue areas (epithelial in brown, mesenchymal in dark green and empty in dark blue) and cell segmentation (cells with separate positivity for Skp2 or Slug are in red and green, respectively; double-positive or negative cells are in yellow or blue, respectively). (**B**) The same area displayed with the cell segmentation only (for both Skp2 and Slug). Separate channels for Skp2 and Slug are provided in (**C**) and (**D**), respectively. All images are at magnification ×200. The analysis found 5.5% double-positive cells in 5 fields of view (9.7% in the area displayed) in the prostate cancer tissue of the patient #62 (pT3a, pN1, Gleason score 4 + 3, serum PSA 2.78 ng/mL), which was in good concordance with the results obtained by standard immunohistochemistry (10 % Skp2 positive and 30 % Slug positive).

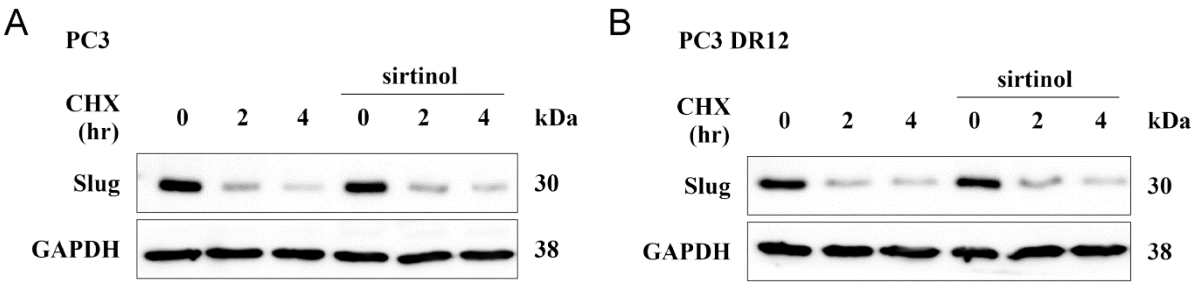

Supplementary Figure S3. Inhibition of deacetylation does not modulate Slug expression. PC3 (**A**) and docetaxel-resistant PC3 DR12 (**B**) cells were pre-treated with sirtinol for 24 hours and then were subsequently treated by 50 µg/mL of CHX (cycloheximide) to prevent de novo protein synthesis. Immunoblots of Slug and GAPDH protein levels are shown at indicated time intervals (hr, hours).

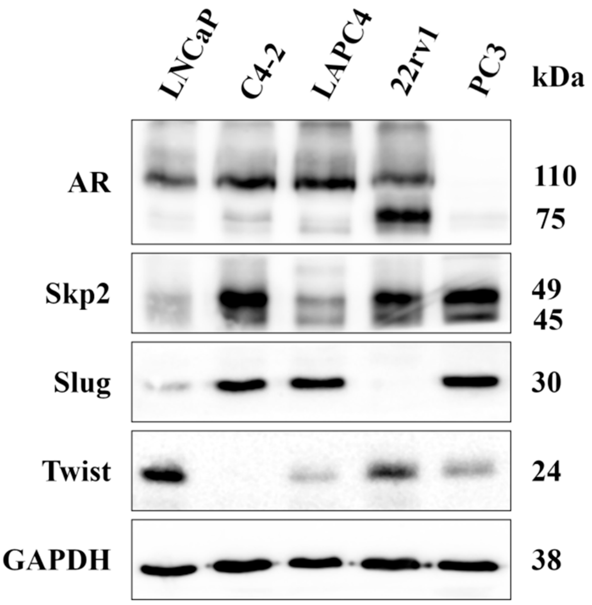

Supplementary Figure S4. Expression profile of androgen-receptor (AR) positive prostate cancer cell lines (LNCaP, C4-2, LAPC-4 and 22rv1) in comparison to the androgen-receptor negative PC3 cells.

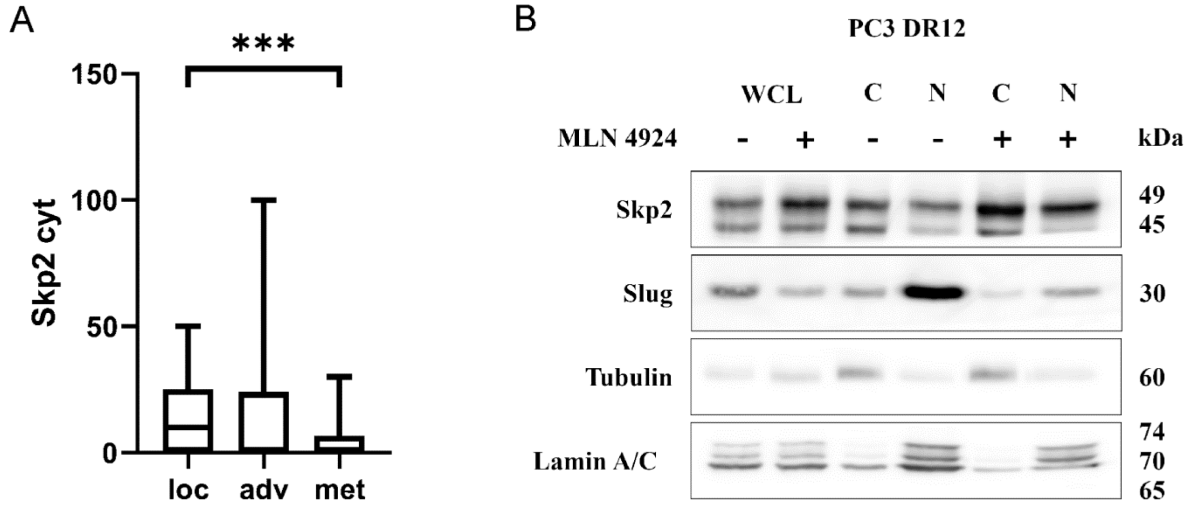

Supplementary Figure S5. Skp2 is present also in the cytoplasm of cancer cells. (**A**) Cytoplasmic expression of Skp2 was significantly lower in patients with lymph node metastasis. Box-plot represents median, 25–75% percentiles, and range of values. *p*-values < 0.05, < 0.01, and < 0.001 are indicated by *, **, and ***, respectively. Median of the cytoplasmic Skp2 expression was lower than the nuclear one (histoscore 5 and 15, respectively; $p < 0.001$; see also Figure 1A). (**B**) Cytoplasmic and nuclear expression of Skp2 and predominant expression of Slug in docetaxel-resistant PC3 DR12 cells after one-day treatment with 2 μM concentration of MLN4924 (WCL, whole cell lysate; N, nuclear; C, cytoplasmic cell fraction).
